# A Systematic Study on Transit Time and Its Impact on Accuracy of Concentration Measured by Microfluidic Devices

**DOI:** 10.3390/s20010014

**Published:** 2019-12-18

**Authors:** Yushan Zhang, Tianyi Guo, Changqing Xu

**Affiliations:** 1 School of Biomedical Engineering, McMaster University, Hamilton, ON L8S 4L7, Canada; zhang749@mcmaster.ca; 2 Forsee Instruments Ltd., Hamilton, ON L8P 0A1, Canada; guotianyi@gmail.com; 3 Department of Engineering Physics, McMaster University, Hamilton, ON L8S 4L7, Canada

**Keywords:** microfluidic, transit time, amplitude, particle/cell concentration counting, accuracy of measurement

## Abstract

Gating or threshold selection is very important in analyzing data from a microflow cytometer, which is especially critical in analyzing weak signals from particles/cells with small sizes. It has been reported that using the amplitude gating alone may result in false positive events in analyzing data with a poor signal-to-noise ratio. Transit time (τ) can be set as a gating threshold along with side-scattered light or fluorescent light signals in the detection of particles/cells using a microflow cytometer. In this study, transit time of microspheres was studied systematically when the microspheres passed through a laser beam in a microflow cytometer and side-scattered light was detected. A clear linear relationship between the inverse of the average transit time and total flow rate was found. Transit time was used as another gate (other than the amplitude of side-scattering signals) to distinguish real scattering signals from noise. It was shown that the relative difference of the measured microsphere concentration can be reduced significantly from the range of 3.43%–8.77% to the range of 8.42%–111.76% by employing both amplitude and transit time as gates in analysis of collected scattering data. By using optimized transit time and amplitude gate thresholds, a good correlation with the traditional hemocytometer-based particle counting was achieved (R^2^ > 0.94). The obtained results suggest that the transit time could be used as another gate together with the amplitude gate to improve measurement accuracy of particle/cell concentration for microfluidic devices.

## 1. Introduction

Microflow cytometers or microfluidic devices for particle/cell detection have been widely investigated and studied in recent years. A combination of electrical [1], mechanical [2], and optical properties such as side-scattered light [3] and laser induced fluorescence [4] have been measured to identify and differentiate the particles/cells. Recently, the physical property of transit time has also been introduced as an indicator for single cell identification using microfluidic devices [1,5]. Cells are squeezed through a constriction region with cell elongation, and the transit time can be recorded to differentiate cells of different sizes. Chen et al. [1] reported that a classification success rate of 93.7% for osteoblasts and osteocytes was achieved by using both transit time and impendence amplitude ratio as threshold gates in data analysis, while the classification success rate was 85.3% if impendence amplitude threshold was used alone.

The majority of the reported microflow cytometers have been used to measure the intensity of either forward scattered light (FSC) or side-scattered light (SSC), as well as the fluorescence light intensity. In some studies, transit time of particles/cells passing through an interrogation region was also measured and used as a gate to filter noise in data processing for particle/cell counting based on a microflow cytometer. Kennedy et al. applied a time-domain filter of 5 µs to filter the raw data collected by a microflow cytometer and pulse duration was also studied [6]. Guo et al. used transit time to differentiate background noise and beads SSC signals from beads [7]. Transit time versus the magnitude of impendence in an impendence microflow cytometer was demonstrated by Bernabini et al. [8]. Transit time of blood cells passing through in capillary-like microchannels was studied for hematologic diseases [9]. Translocation profiles of cells passing through micropores were applied to identifying tutor cells and blood cells [10], and Ali et al. created a solid-state micropore device capable of distinguishing metastatic and non-metastatic tumor cells based on the translocation time and amplitude profiles [11]. Although transit time has been introduced in the data analysis of microflow cytometers, a systematic study on transit time and its impact on the performance of a microflow cytometer has not yet been reported. Questions such as how the transit time depends on measurement conditions remain unanswered. 

The fundamental goal of microflow cytometers is to count and group cells/particles precisely [12]. Setting proper thresholds on the collected data is critically important to data analysis for microflow cytometers [13,14,15,16,17,18]. This paper presents a systematic study on transit time in processing data collected from a microflow cytometer under different measurement conditions. The effects of transit time on the accuracy of microsphere counting were investigated. An optimized transit time threshold setting method was provided to increase the accuracy of the microsphere counting. Moreover, the results of the microflow cytometer were compared to those based on the traditional hemocytometer counting.

## 2. Materials and Methods

### 2.1. Materials 

The materials used in the measurements include polystyrene microspheres, SU-8 photoresist, and the Sylgard 184 Silicone Elastomer Kit. Polystyrene microspheres were purchased from Polysciences Inc. (Warrington, PA, USA), SU-8 photoresist from MicroChem Corp (Newton, MA, USA), and Sylgard 184 Silicone Elastomer Kit from Dow Corning (Midland, MI, USA).

### 2.2. Microflow Cytometer 

**Figure 1:**
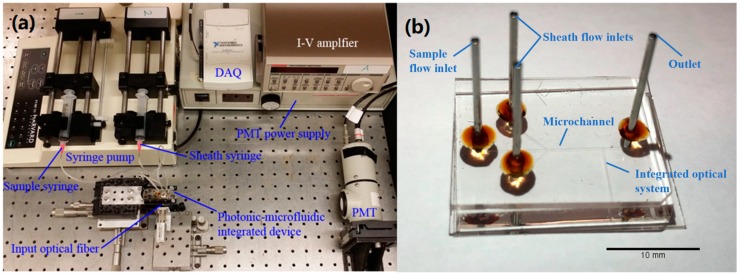
(**a**) A photo of the system setup of the microflow cytometer. (**b**) The microfluidic device.

The core element of the microflow cytometer is an optofluidic device fabricated by the standard photolithography and soft lithography techniques, as shown in Figure 1. The detailed fabrication process has been described in previous publications [3,7]. A 2D hydrodynamic focusing channel with two inlets and one outlet was fabricated by SU-8 (50 µm × 100 µm, SU-8 2075) and the channel was sealed by Polydimethylsiloxane (PDMS) using nitrogen plasma treatment [19]. An on-chip lens system was designed to guide and focus the excitation laser beam with a waist of 10 µm. 

### 2.3. Sample Preparation

Polystyrene microspheres with a diameter of 2 µm were suspended in deionized water and a 3-fold serial dilution of the microspheres was performed, resulting in a serial of samples with microsphere concentration in a range between 1 × 10^6^ and 6 × 10^3^ microspheres/mL. The microsphere concentrations of the diluted samples were measured by both a hemocytometer under a microscope and the microflow cytometer more than three times.

In all microsphere experiments, surfactant (0.1% SDS) was added to reduce hydrophobicity. The samples in the test tubes were mixed on a vortex mixer and then underwent a sonication process to disrupt aggregates before use. The total flow rate was controlled under 16 mL/h to make sure the microspheres were distributed uniformly along the flow direction. 

### 2.4. Data Collection and Analysis

Side-scattered light signals of the microspheres were collected by a photomultiplier tube (PMT) connected to a data acquisition card (DAQ) with a sampling frequency of 250 kHz. An automatic data collection and analysis platform for the microflow cytometer was designed and implemented by a custom-made LabView (National Instruments) program on a computer.

## 3. Results and Discussion

### 3.1. Microflow Cytometer Data Analysis

**Figure 2:**
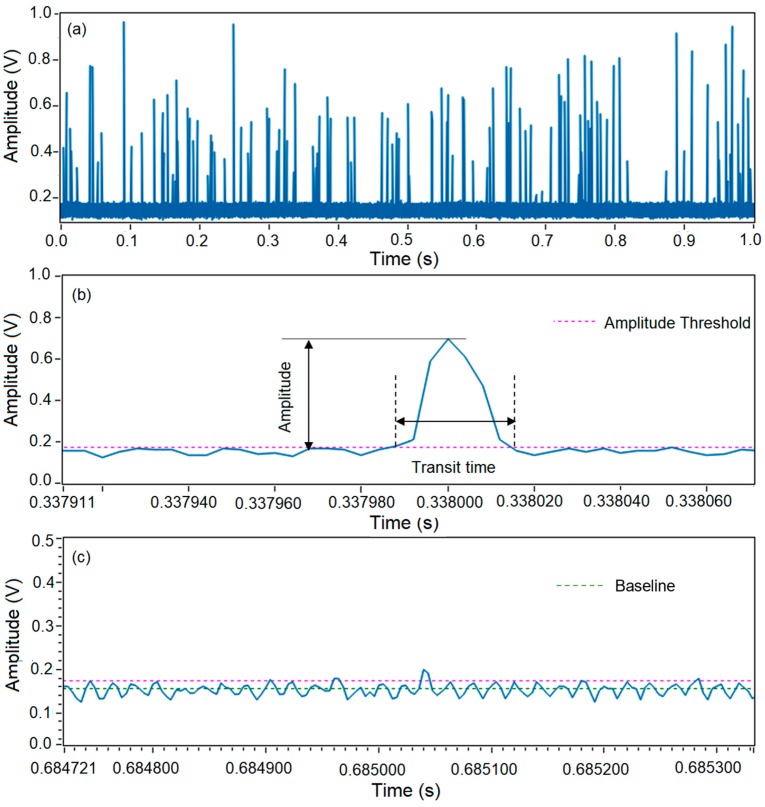
Data collection and analysis of a microsphere test using a microflow cytometer. (**a**) Raw data of 1 s from a test with a duration of 60 s. (**b**) Amplitude and transit time read out from a positive event. (**c**) Amplitude and transit time read out form a negative event.

Microspheres were injected into the microflow cytometer at a flow rate of 1500 µL/h and a total flow rate of 6000 µL/h. Figure 2a shows one second of raw data of SSC signal from a test with a duration of 60 s. Large spikes generated by microspheres and small spikes generated by background noise can be observed. Clearly, an amplitude threshold needs to be set to differentiate the positive events representing microspheres and false events representing the noise. Figure 2b shows an enlarged plot of raw signals in Figure 2a from 0.337911 to 0.338060 s. This pulse has a peak of 0.72 V, and thus can easily be picked up and counted as a positive event. In this case, an amplitude threshold of 0.18 V was applied, which was four times of the standard deviations above the baseline.

However, some pulses may not be created from microspheres even though amplitudes of these pulses are above the amplitude threshold, as shown in Figure 2c. The majority of the noise was under 0.18 V and was eliminated by the amplitude threshold, while a pulse of 0.21 V was considered as a positive event as it was 0.03 V greater than the threshold in Figure 2c. Based on empirical data, this pulse resulted from noise and it would cause a false positive event. A common characteristic of this noise is that their amplitudes were only a little bit larger than the amplitude threshold. Although this noise could be removed by setting a higher amplitude threshold, some weak pulses generated by microspheres not located in the center of the microchannel could be rejected as well. As a result, it is impossible to eliminate these false counts simply by applying an amplitude threshold. 

As shown in Figure 2b, pulses resulted from positive events were tall and wide, i.e., high amplitude and long pulse duration. By contrast, the majority of pulses resulting from noise were weak and short (see Figure 2c). Therefore, it would be helpful to introduce a time-domain filter as an additional gate to the amplitude gate. Transit time is a natural choice for the time-domain filter. Transit time can be defined as the time period between the two cross-points of a pulse and the amplitude threshold, which can be read out from the raw data. Based on this definition, the transit time of the pulse in a positive event in Figure 2b was read as 29.4 µs, which matches well with the calculated time of 30 µs for the microspheres running through the optical beam in the experiments (average fluid velocity in the microchannel was 0.33 m/s, and the beam waist was 10 µm). By contrast, the transit time read from the false positive event in Figure 2c was about 8 µs, which is much shorter than the average transit time. Therefore, the pulse with a transit time of 8 µs shown in Figure 2c can be rejected by employing a transit time gate. 

**Figure 3:**
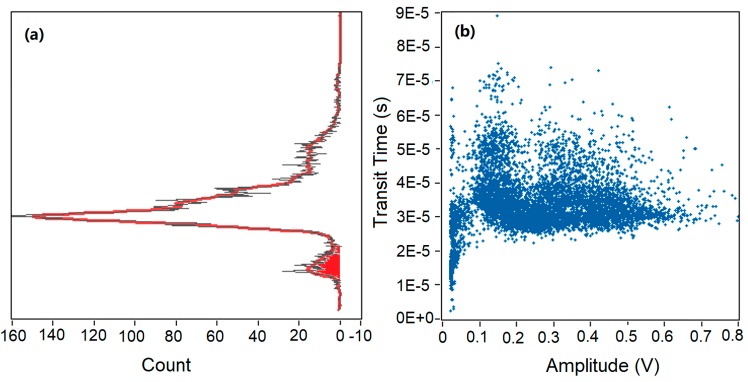
Data analysis of 60 s raw data. (**a**) Transit time distributions based on counts. (**b**) Scattering plot of all events based on transit time and relative amplitude above amplitude threshold.

As shown in Figure 3, the transit time and amplitude of every pulse can be read out from the raw data, and every pulse can be counted as an event. In this way, every event can be described by using both transit time and amplitude. Raw data of 60 s collected in Figure 2 is analyzed and plotted in Figure 3. In Figure 3a, the x-axis represents the number of events and the y-axis represents the transit time extracted from each event. Figure 3b shares the same y-axis with Figure 3a, and the x-axis represents the amplitude above the amplitude threshold. It is shown that the obtained profile of amplitude in Figure 3b is not apparently sharp enough to distinguish false events from positive events as discussed earlier in Section 3.1. If a higher amplitude threshold was applied in the data processing as amplitude threshold moved to the right of the y-axis in Figure 3b, some noise would be rejected, and some positive events generated by the microspheres would be rejected as well. If a lower amplitude threshold was set as the amplitude threshold moved to the left of the y-axis in Figure 3b, more noise would be included. Due to the limitation of the amplitude gating, another gate is needed to solve the problem.

In Figure 3a, a fitting curve (red) of counts versus transit time was achieved. One maximum peak of counts and a sub-peak were found in Figure 3a, corresponding to a transit time of 30 µs and 8 µs, respectively. Interestingly, similar peaks and sub-peaks were found in all experiments during the data analysis process. It can be observed that a majority of events had a transit time range of 25–40 µs, and a maximum count occurred at a transit time of 30 µs. As mentioned above, the estimated average transit time for the microspheres passing through the laser beam was also 30 µs. Thus, we can conclude that the maximum peak resulted from the microspheres and was corresponded to real events. In addition, a wide spread of transit time (0–75 µs) was found in Figure 3. Transit time varied from 10 to 22 µs in the red region in Figure 3a, and some events had a longer transit time (40–75 µs) above the average transit time. The wide spread of transit time was caused by the parabolic velocity profile in the 2D hydrodynamic focused microchannel. Microspheres were focused in the center of the microchannel along the flow direction, while they were distributed quadratically in the direction perpendicular to the flow. 

Microspheres focused in the center of the channel passed through the interrogation region at a higher velocity and a shorter transit time, and microspheres distributed at the top and bottom of the microchannel had a lower velocity and a longer transit time. As we can see from Figure 3, a clear boundary between the maximum peak and the boundary peak was found based on the transit time. Thus, a transit time threshold was set at 22 µs to reject the background noise. Events in the red region of the sub-peak had a shorter transit time and a small amplitude above threshold. These events were considered as positive events by amplitude threshold alone, and they were rejected by applying the time-domain filter. Amplitude threshold alone was not able to eliminate the sub-peak caused by background noise, and transit time was a powerful tool for removing false counting. Therefore, it is necessary to use both amplitude and transit time thresholds to analyze the SSC data in microfluidic devices.

### 3.2. The Dependence of Inverse Transit Time on Total Flow Rate

**Figure 4:**
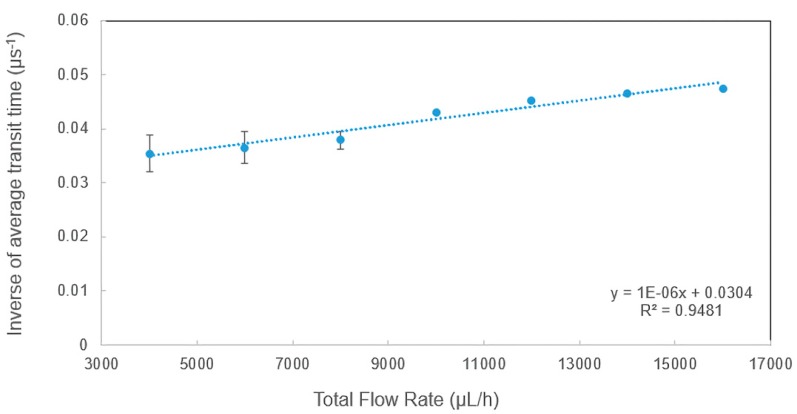
The dependence of inverse of average transit time on total flow rate.

To study dependence of the transit time on measurement conditions, a detailed study on transit time was performed and the raw data were analyzed using the methods explained in Section 3.1. Figure 4 shows the measured transit time under various total flow rates. In the experiments, the total flow rate was increased from 4000 to 16,000 µL/h while the sheath flow rate to sample flow rate ratio was kept constant at three in order to maintain a good hydrodynamic focusing. As shown in Figure 4, the inverse transit time (τ_avg_^−1^) had a very good linear relationship with the total flow rate (Q), which can be described well by the simplified equation below, where τ_avg_^−1^ is the inverse of average transit time, L is the interrogation width and A is the cross-sectional area of the microchannel.

$${\tau_{\mathrm{avg}}}^{-1}=\frac{Q}{L*A}$$

The average velocity can be determined by the ratio of the total flow rate (Q) to the cross-sectional area (A), and it is also equal to the interrogation width over average transit time. Figure 4 indicates that the velocity depends on the flow rate and the linear relationship verified the performance of the microflow cytometer. The y-intercept of 0.0304 (µs^−1^) was due to system error. As the scattered light can be produced wider than the beam waist in real experiments, the slope of the linear relationship was close to but not exactly equal to 1/(L*A). 

It is worth noting that the average transit time at 6000 µL/h extracted from the raw data shown in Section 3.2 matches well with the calculated average transit time. With the increase of total flow rate, the velocity of the microspheres is increased, and the transit time is decreased. Therefore, the inverse transit time had a linear relationship with the total flow rate. Similar results were also found in experimental tests using microspheres of varying sizes (e.g., 6 µm). As the diameters of the microspheres increase, the average transit time will be longer, and the amplitude of the side-scattered light will be stronger. 

### 3.3. Comparison between the Traditional Counting and the Microflow Cytometer

**Figure 5:**
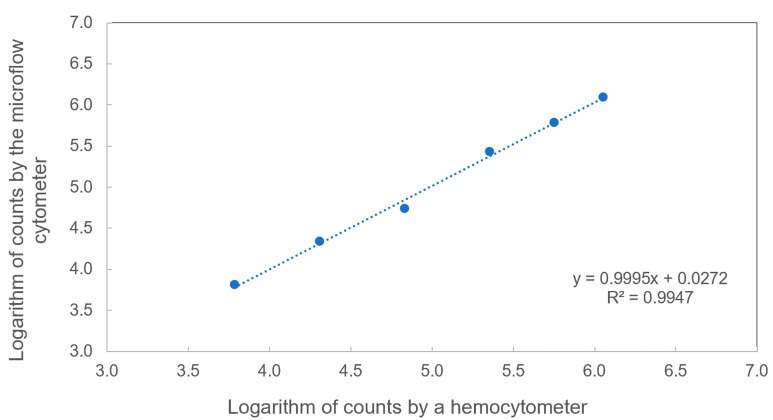
Comparison between two different methods. Microspheres were measured both by a hemocytometer and a microflow cytometer. The performance of the optofluidic microflow cytometer exhibited a linear response to hemocytometer counts on a logarithmic scale.

To verify the particle counting accuracy of the microflow cytometer, particle counting based on the traditional hemocytometer and the microflow cytometer was conducted, as shown in Figure 5. A 3-fold serial dilution based on an initial sample with a concentration of 1.14 × 10^6^ microspheres/mL was performed to get six microsphere samples. The microsphere samples ran through the microflow cytometer for a 60 s test under the same experimental conditions described in Section 3.1, and the raw data were analyzed by applying both transit time and amplitude thresholds. At the same time, these samples were also counted under a microscope using a hemocytometer more than three times. The measured concentrations of these samples by the two methods were plotted on a logarithmic scale and fitted by a linear curve in Figure 5. A linear fitting with a R^2^ value greater than 0.999 was achieved, and the relative standard deviation of the concentrations measured by the microflow cytometer was within a range of 0.99% to 4.73%, implying that the microflow cytometer has the capability of counting microspheres with a high accuracy over a wide concertation range of 6.13 × 10^3^ to 1.14 × 10^6^ microspheres/mL.

### 3.4. The Accuracy of Transit Time Threshold

**Figure 6:**
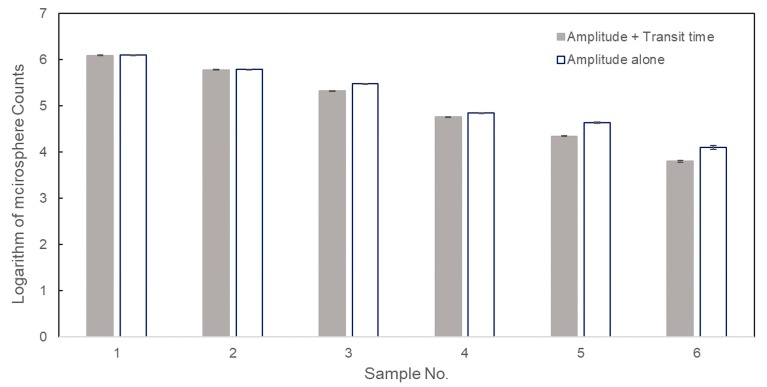
Logarithmic counting results of six microsphere samples using amplitude and transit time thresholds, and amplitude threshold alone.

Although Figure 5 demonstrated the results achieved from the microflow cytometer can be trusted, the impact of the transit time threshold on measurement accuracy of concentration still needs to be figured out. The raw data obtained in Section 3.3 were processed with and without setting of a transit time threshold and the logarithm of microspheres counting results for six samples were plotted in Figure 6. The standard deviations of tests without a transit time threshold ranged from 0.0072 to 0.043, while the values were in a range of 0.0058 to 0.018 in tests with a transit time threshold. Standard deviations can also be decreased by the transit time threshold, and these low standard deviations can also verify the good repeatability of the microflow cytometer. As shown in Figure 6, no significant differences between the two data analysis methods were shown when the concentration was high (samples 1 and 2). This result can be explained in that the false positive events caused by the background noise can be ignored due to the large number of positive events generated by microspheres at high sample concentrations, and it has validated our discussion in Section 3.1. As microsphere concentration was lower than 10^6^ microspheres/mL, the measured results using amplitude threshold alone were greater than the results measured by employing both amplitude and transit time thresholds (samples 3–6 in Figure 6). This is because the pulses generated by background noise become comparable to the pulses created by microspheres as the microsphere concentration declines from 1.14 × 10^6^ to 6.13 × 10^3^ microspheres/mL. Therefore, eliminating false positive events by setting a transit time threshold is especially important to measurement accuracy at low concentration or at low microsphere throughput. 

Table 1 shows relative difference in percentage using amplitude threshold alone and using both transit time and amplitude thresholds. The relative difference in Table 1 is defined as the ratio of difference between the concentrations measured by the microflow cytometer and the values measured by a hemocytometer. When the concentration of the microspheres dropped to 10^3^–10^4^ microspheres/mL, the relative difference was as large as 108%–111% without setting a transit time filter. These results suggest that the transit time threshold can increase the accuracy of the measured microsphere concentration especially at low concentrations. 

## 4. Conclusions

This paper presented a systematic study on transit time and its impact on microsphere concentration detection. We have demonstrated how the transit time threshold can be set under various measurement conditions. Investigation of data analysis with or without setting a transit time threshold has been performed to evaluate its impact on the accuracy of the measurement. It has been found that the inverse of the average transit time depends on the total flow rate and the transit time can be used as a second gate to further differentiate real signals from background noise after filtering by the amplitude threshold. The measured accuracy has been improved significantly by applying both transit time and amplitude thresholds, which is especially important at low microsphere concentrations. The relative differences achieved without transit time gating ranged between 9.65%–111.76%, while these values dropped significantly to 3.43%–8.77% by employing both amplitude and transit time gating. The measured concentrations can be still reliable and accurate at lower concentrations (~10^3^ microspheres/mL). At the same time, a linear fitting over a wide range of microsphere concentrations has been achieved between the microflow cytometer method and the traditional hemocytometer method. It has been shown that transit time as a gate for microflow cytometers has the capability of enhancing the accuracy of the side-scattered light signal detection method, and it could be applied to other microfluidic devices for particle/cell detection as well. 

## Figures and Tables

**Table 1:** Differences in accuracy of methods with and without transit time gating for counting microspheres.

Sample	Relative Difference with Transit Time Gating (%)	Relative Difference without Transit Time Gating (%)
1	8.77	9.65
2	6.67	8.42
3	−6.61	32.60
4	−6.96	12.46
5	7.84	111.76
6	3.43	108.81

## References

[1] Chen J., Zheng Y., Tan Q., Shojaei-baghini E., Zhang Y., Li J., Prasad P., You L., Wu X., Sun Y. (2011). Classification of cell types using a microfluidic device for mechanical and electrical measurement on single cells. Lab Chip.

[2] Hou H.W., Li Q.S., Lee G.Y.H., Kumar A.P. (2009). Deformability study of breast cancer cells using microfluidics. Biomed. Microdevices.

[3] Guo T., Wei Y., Xu C., Watts B.R., Zhang Z., Fang Q., Zhang H., Selvaganapathy P.R., Deen M.J. (2015). Counting of Escherichia coli by a microflow cytometer based on a photonic-microfluidic integrated device. Electrophoresis.

[4] Xun W., Feng J., Chang H. (2015). A Microflow Cytometer Based on a Disposable Microfluidic Chip With Side Scatter and Fluorescence Detection Capability. IEEE Trans. Nanobiosci..

[5] Goddard C.M., Allard M.F., Hogg J.C., Herbertson M.J., Walley K.R. (1995). Prolonged leukocyte transit time in coronary microcirculation of endotoxemic pigs. Am. J. Physiol. Heart Circ. Physiol..

[6] Kennedy M.J., Stelick S.J., Sayam L., Yen A., Erickson D., Batt C.A. (2011). Hydrodynamic optical alignment for microflow cytometry. Lab Chip.

[7] Guo T. (2016). An Optical System towards In-line Monitoring of Bacteria in Drinking Water. Ph.D Thesis.

[8] Bernabini C., Holmes D., Morgan H. (2011). Micro-impedance cytometry for detection and analysis of micron-sized particles and bacteria. Lab Chip.

[9] Rosenbluth M.J., Lam A., Fletcher D.A. (2008). Analyzing cell mechanics in hematologic diseases with microfluidic biophysical flow cytometry. Lab Chip.

[10] Asghar W., Wan Y., Ilyas A., Bachoo R., Kim Y.T., Iqbal S.M. (2012). Electrical fingerprinting, 3D profiling and detection of tumor cells with solid-state micropores. Lab Chip.

[11] Ali W., Ilyas A., Bui L., Sayles B., Hur Y., Kim Y.T., Iqbal S.M. (2016). Differentiating Metastatic and Non-metastatic Tumor Cells from Their Translocation Profile through Solid-State Micropores. Langmuir.

[12] Aghaeepour N., Finak G., Consortium T.F., Consortium T.D., Hoos H., Mosmann T.R., Brinkman R., Gottardo R., Scheuermann R.H. (2013). Critical assessment of automated flow cytometry data analysis techniques. Nat. Methods.

[13] Snow C.K. (2004). Flow cytometer electronics. Cytometry A.

[14] Herzenberg L.A., Tung J., Moore W.A., Herzenberg L.A., Parks D.R. (2006). Interpreting flow cytometry data: A guide for the perplexed. Nat. Immunol..

[15] Jimenez-Carretero D., Ligos J.M., Martínez-López M., Sancho D., Montoya M.C. (2018). Flow Cytometry Data Preparation Guidelines for Improved Automated Phenotypic Analysis. J. Immunol..

[16] Lugli E., Roederer M., Cossarizza A. (2010). Data analysis in flow cytometry: The future just started. Cytometry A.

[17] Pyne S., Hu X., Wang K., Rossin E., Lin T., Maier L.M., Baecher-allan C., Mclachlan G.J., Tamayo P., Hafler D.A. (2009). Automated high-dimensional flow cytometric data analysis. Proc. Natl. Acad. Sci. USA.

[18] Lo K., Brinkman R.R., Gottardo R. (2008). Automated Gating of Flow Cytometry Data via Robust Model-Based Clustering. Cytometry A.

[19] Zhang Z., Zhao P., Xiao G., Watts B.R., Xu C. (2011). Sealing SU-8 microfluidic channels using PDMS. Biomicrofluidics.

